# Toward a treatment for thyroid hormone transporter MCT8 deficiency – achievements and challenges

**DOI:** 10.1530/ETJ-24-0286

**Published:** 2024-11-20

**Authors:** Boyka Markova, Steffen Mayerl, Heike Heuer

**Affiliations:** 1 Department of Endocrinology, Diabetes & Metabolism, University Hospital Essen, University of Duisburg-Essen, Essen, Germany; 2 Center for Translational Neuro- and Behavioral Sciences (C-TNBS), Medical Faculty, University of Duisburg-Essen, Essen, Germany

**Keywords:** thyroid hormone transporter, Allan–Herndon–Dudley syndrome, MCT8, SLC16A2, OATP1C1, SLCO1C1, TRIAC, DITPA, gene therapy, phenylbutyrate

## Abstract

Patients with an inactive thyroid hormone (TH) transporter MCT8 (Allan–Herndon–Dudley Syndrome, AHDS) display severe neurological impairments and motor disabilities, indicating an indispensable function of MCT8 in facilitating TH access to the human brain. Consequently, the CNS of AHDS patients appears to be in a TH deficient state, which greatly compromises proper neural development and function. Another hallmark of this disease is that patients exhibit elevated serum T3 levels, leading to a hyperthyroid situation in peripheral tissues. Several treatment strategies have been developed and evaluated in preclinical mouse models as well as in patients. Here, we discuss these different therapeutic approaches to overcome MCT8 deficiency and summarize the current achievements and challenges in improving brain maturation in the absence of MCT8.

## Introduction

Thyroid hormone (TH) transporters facilitate the transmembrane passage of T4 and T3 and are thus mandatory for proper TH metabolism (by intracellularly localized iodothyronine deiodinases) and TH signaling (by intracellularly localized TH receptors) (1). A vast array of transporters accepting TH as substrates has been identified so far (2, 3). Yet, to what extent these proteins are physiologically relevant for facilitating cellular TH uptake and efflux *in vivo* is still largely unexplored, particularly as most of them are multi-specific and transport a variety of different substrates. Moreover, different cell types may express a distinct repertoire of TH-transporting proteins that is species-specific and can be spatiotemporally altered, as exemplified in studies of brain TH transporter expression patterns (4, 5). Therefore, it is not too surprising that only for a few TH transporter candidates, a (patho-) physiological function has so far been disclosed.

The most specific and best-studied TH transporter to date is the monocarboxylate transporter 8 (MCT8), which is encoded by the X-linked *SLC16A2* gene. MCT8 mediates the transmembrane passage of T3 and T4 via facilitated diffusion and thus can act both as a TH uptake and efflux system depending on the TH concentration gradient. Both in humans and rodents, MCT8 exhibits a widespread distribution pattern with strong expression in the developing and adult brain, liver, kidney, and thyroid, among other tissues (2, 3).

The physiological relevance of MCT8 became evident with the identification of patients with affected MCT8 function, also known as Allan–Herndon–Dudley Syndrome (AHDS) (OMIM number 300523) (6, 7, 8). To date, more than 150 different mutations in the human *SLC16A2* gene have been discovered that mitigate or even completely abolish MCT8-mediated TH transport function (9). The predominant clinical symptoms of this rare disease (with an estimated prevalence of 1:70,000 males) comprise severe intellectual and motor disabilities, suggesting an insufficient TH transport across brain barriers and into neural cells (10, 11). As another hallmark of MCT8 deficiency, affected patients display deranged TH serum parameters with elevated T3, low/normal T4, and high/normal TSH together with symptoms (e.g. low body weight, muscle wasting, and tachycardia) indicating a hyperthyroid state in several peripheral tissues. In essence, the clinical picture of AHDS, which is described in-depth elsewhere (12, 13), includes clinical signs of peripheral thyrotoxicosis and central TH deficiency and is thus very unique. Brain TH deprivation in AHDS is considered to be primarily caused by an abrogated TH transport across blood–brain barrier (BBB) cells. This assumption is supported by histomorphological studies of patient-derived brain samples, as these analyses disclosed altered brain maturation parameters well known from animal models with impaired TH signaling (10, 14). Yet, the exact spatiotemporal disturbances that occur during brain development in MCT8 deficiency are far from being fully understood.

## Preclinical model systems

The generation of several Mct8-deficient animal models (e.g. mouse, chicken, and zebrafish) has underscored the relevance of MCT8 in facilitating TH access to the CNS and into neural cells (2). Yet, none of the available model systems fully mirrors all aspects of human MCT8 deficiency, most likely due to species-specific differences in spatiotemporal TH transporter expression patterns. Global as well as cell-specific Mct8 knockout (KO) mice turned out to be highly informative for studying Mct8 function outside the CNS. Notably, they were most useful to disclose the pathological events that ultimately cause the characteristic abnormal serum TH profile (15). However, brain development and function in global Mct8 KO mice turned out to be surprisingly normal as T4 passage to the Mct8-deficient murine CNS is only reduced by 50% (16, 17). This latter observation has led to the discovery of the organic anion transporter Oatp1c1 as an alternative and physiologically relevant T4 transport system in the murine CNS (18, 19, 20). With T4 still being available for local T3 conversion in the Mct8 KO CNS, a compensatory rise in astrocytic type 2 deiodinase (Dio2) appears to prevent severe brain TH deprivation and brain damage in Mct8 KO animals (16, 21).

Murine Oatp1c1 (encoded by the *Slco1c1* gene) exhibits a very restricted distribution pattern with the highest expression in murine BBB and blood–cerebrospinal fluid barrier (BCSFB) structures (4). Intriguingly, OATP1C1 is not expressed in human BBB endothelial cells and thus cannot compensate for a non-functional MCT8 in AHDS patients (22, 23). However, the human OATP1C1 protein has been localized to neurons of basal ganglia circuits (24) and single cell transcriptome analysis revealed human SLCO1C1 mRNA expression in radial glia cells and astrocytes, suggesting that this transporter fulfills specific functions also in the human CNS during fetal development (25). The latter hypothesis is supported by clinical findings in one juvenile patient with a biallelic mutation in OATP1C1, who was diagnosed with dementia, white matter degeneration, and severe glucose hypometabolism (26). However, to what extent this clinical phenotype can be attributed to impaired T4 transport in distinct cell types is still a matter of debate.

**Figure 1:**
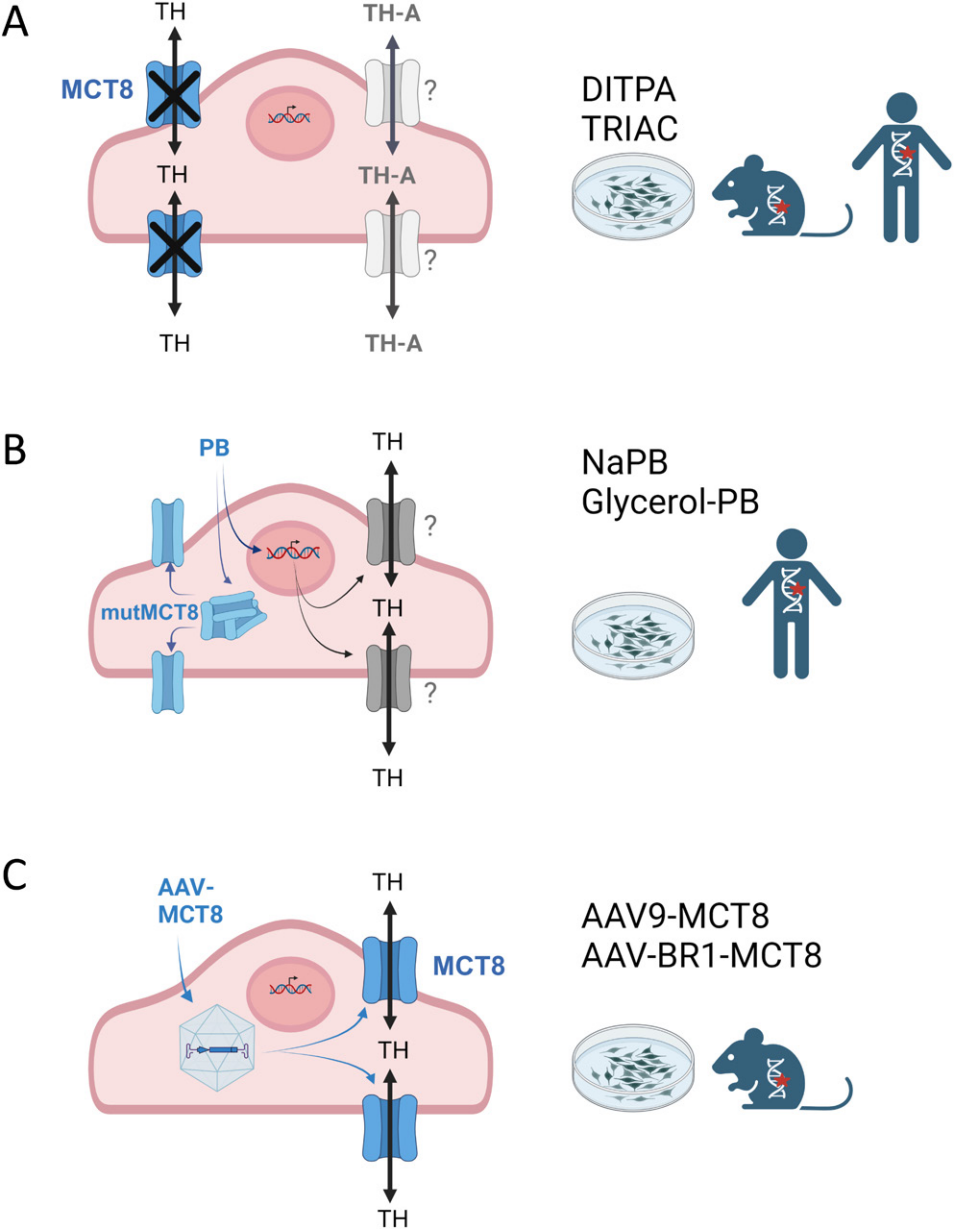
Different approaches to overcome MCT8 deficiency at brain barrier cells. (A) Thyroid hormone analogs (TH-A) such as DITPA or TRIAC are transported across cell membranes independent of MCT8 and can exert thyromimetic action in neural cells. (B) Treatment with the chaperone phenylbutyrate (PB) improves surface translocation of misfolded MCT8 and also stimulates the expression of other TH transporters. (C) Application of adeno-associated virus (AAV)-MCT8 vector constructs targeting blood-brain barrier cells restores MCT8 protein expression and, consequently, TH transport into the CNS.

Mice with a combined deletion of Mct8 and Oatp1c1 exhibit a profound state of TH deficiency in the CNS due to strongly abolished T3 and T4 transport across brain barrier cells (27). Mct8/Oatp1c1 double-knockout (DKO) mice display locomotor disabilities together with distinct morphological alterations in neural maturation that can be considered hallmarks of impaired central TH signaling. In particular, delayed cerebellar maturation, a reduced number of inhibitory parvalbumin (PV)-expressing cortical interneurons, and hypomyelination are characteristic features of a TH-deficient mouse CNS (28, 29, 30, 31). A similar situation can be achieved by combining Mct8 KO mice with Dio2-deficient animals in order to prevent compensatory astrocytic T3 production in Mct8 deficiency. These Mct8/Dio2 DKO mice exhibit impaired motor skills, hypomyelination, and a disturbed GABAergic circuit system, making them also suitable as a mouse model for AHDS (32, 33). It is important to note that both Mct8/Oatp1c1 and Mct8/Dio2 double-knockout (DKO) mice have their distinct limitations. As Oatp1c1 exhibits a broader substrate specificity than Mct8 (34), a functional Oatp1c1 at the murine BBB may be required to transport other CNS-active compounds that affect brain maturation independent of TH. Mct8/Dio2 DKO mice, in turn, exhibit strongly reduced brain T3 concentration due to abolished astrocytic T3 production, but in contrast to Mct8/Oatp1c1 DKO mice, they do not replicate the drop in brain T4 content seen in AHDS (14, 32). Moreover, though both mouse models certainly do not fully recapitulate the severe neurological and motor disabilities of AHDS, they have been very useful to evaluate putative treatment options and to test therapeutic strategies. In the following paragraphs, we will shortly summarize the current preclinical and clinical approaches that have so far been developed and evaluated (Fig. 1)

## L-T4 and L-T4/PTU treatment

MCT8-deficient patients were empirically treated with L-T4 under the suspicion of congenital (central) hypothyroidism (2). However, it became evident that L-T4 treatment of affected patients neither improved neurological function nor normalized thyroid function test parameters. Instead, it resulted in an even further rise in serum T3, with negative outcomes on metabolism and body weight (2). Though this response was initially unanticipated, the underlying reasons became obvious upon phenotyping Mct8-deficient mice. These studies revealed that the rise in serum T3 is caused by alterations in TH production and metabolism in peripheral tissues. In the Mct8-deficient thyroid gland, T4 efflux is compromised, leading to a reduced thyroidal T4 secretion despite elevated intrathyroidal T4 and T3 synthesis (35, 36). Moreover, a striking accumulation of T4 in the Mct8-deficient kidneys indicates a critical function of Mct8 as a renal T4 efflux system in proximal tubule cells (16). A profound increase in renal and hepatic type 1 deiodinase (Dio1) activities and, consequently, a strong rise in renal and hepatic T3 production appears to underpin the elevated serum T3. This assumption is substantiated by the rather normal T3/T4 ratio found in mice lacking both Mct8 and Dio1 (37). The latter finding clearly underscores the pathogenic relationship between elevated Dio1 activities and increased serum T3 levels. Furthermore, these observations suggest that the exogenously applied L-T4 given to the AHDS patients was metabolized immediately to T3 by increased peripheral DIO1 activities, thus worsening the peripheral thyrotoxicosis.

These preclinical findings have been successfully translated into clinical practice. By treating AHDS patients with L-T4 in combination with the DIO1 inhibitor PTU, a normalization of serum T4 and T3 parameters could be achieved (38, 39). Moreover, under L-T4/PTU combination therapy, peripheral thyrotoxic symptoms significantly ameliorated and patients gained more weight. Yet, due to its short half-life, PTU has to be constantly applied three times per day to inhibit DIO1 effectively and can also provoke severe side effects such as agranulocytosis and liver damage. Thus, such a treatment regimen is not feasible for long-term therapy. Even more importantly, L-T4/PTU combination therapy is not expected to exert any beneficial impact on CNS function as long as TH uptake into the CNS remains impaired in AHDS.

## Treatment with thyromimetic substances

In order to improve the apparent TH-deficient state in the CNS in MCT8 deficiency, the application of thyromimetic substances, such as 3,5-diiodothyropropionic acid (DITPA) and 3,3’,5-tri-iodothyroacetic acid (TRIAC; tiratricol), was considered based on their capability to enter cells independent of MCT8 and was extensively explored in (pre-)clinical studies (40, 41). Finally, the synthetic thyromimetic sobetirome (GC-1) and its BBB-penetrating prodrug Sob-AM2 have recently been tested in fetal and adult Mct8-deficient mice (42, 43, 44). Yet, to what extent these latter compounds improve brain maturation and reduce peripheral thyrotoxicosis in Mct8 deficiency still needs to be explored.

DITPA represents a synthetic TH analog with low metabolic activity that binds TH receptors (TR) alpha and beta equally well. Yet, the affinity of DITPA toward TRs is around 350-fold lower compared to T3; hence, high concentrations of DITPA are required to achieve thyromimetic effects (45). Treatment of adult Mct8 KO mice with DITPA indeed reduced hepatic Dio1 activities (possibly by inhibiting the enzyme) and improved symptoms of peripheral thyrotoxicosis but seemingly did not affect TH-regulated gene expression in the CNS (46). In contrast, DITPA daily injected into Mct8/Oatp1c1 DKO animals during the first three postnatal weeks restored cerebellar maturation and myelination, indicating that this compound can indeed exert thyromimetic functions in the mouse CNS during early postnatal stages (47). Yet, a compassionate DITPA treatment of four MCT8 patients initiated at the age of 8–25 months did not result in any significant amelioration of CNS symptoms, though this treatment was successful in correcting abnormal thyroid function test parameters and peripheral hypermetabolism (48). Whether a neonatal initiation of DITPA in patients would have a greater impact on CNS maturation still needs to be tested.

In contrast to DITPA, TRIAC represents a natural TH metabolite with a similar affinity for TR alpha1 and an even three- to six-fold higher affinity for TR beta compared to T3, and thus exerts a strong TSH suppressive effect (45). This action has already been extensively explored in clinical practice for the treatment of thyroid carcinoma and RTH beta patients, as reviewed elsewhere (12, 49). Treatment of neonatal Mct8/Oatp1c1 DKO mice with TRIAC during the first three postnatal weeks turned out to be highly effective in restoring neural maturation as well as locomotor performance (47). Early onset TRIAC treatment of DKO mice also resulted in normalized functional connectivity in fMRI studies, underscoring the robust and long-lasting beneficial effects of TRIAC on postnatal brain maturation and function (50). However, the exact time point of treatment initiation appears to be very critical. TRIAC application to mice after weaning had very little impact on brain parameters, as shown in different models and routes of application (47, 51, 52).

Why are the thyromimetic effects of TRIAC on murine brain development limited to the early postnatal period? It has been speculated that potential transporter proteins enabling TRIAC access to the brain and into neural cells might be downregulated with age. This hypothesis is supported by the recent discovery of several human and murine TRIAC-transporting proteins (53, 54). Particularly, the Slc22a8 transporter present at the murine BBB and BCSFB is strongly expressed only during the early postnatal phase (53, 54). Alternatively, the window of opportunity to restore TH-dependent CNS maturation processes might close in mice after the first postnatal weeks, and thus neither DITPA nor TRIAC administration to the juvenile AHDS mouse model exerts any beneficial outcome on neural cells.

Application of TRIAC to AHDS patients has been assessed in a single-arm, open-label, phase 2 clinical study. In this TRIAC Trial I (NCT02060474), 42 pediatric and adult MCT8 patients were treated for 12 months with TRIAC to evaluate its impact on peripheral parameters and to unravel potential side effects (55). The outcome of this trial confirmed the safety of the intervention. Moreover, a consistent reduction of the high serum T3 levels together with positive effects on body weight, cardiac, and metabolic parameters could be achieved, advocating for TRIAC as a suitable treatment to ameliorate the symptoms of peripheral thyrotoxicosis (55, 56). Due to the wide age range of the patients enrolled in this trial, neurocognitive alterations were only evaluated in an exploratory way and disclosed a trend of neurodevelopmental improvement in those patients who received TRIAC early in life. A second international phase IIb trial (TRIAC Trial II; NCT02396459) is currently being conducted to assess the neurocognitive outcome in MCT8 patients who are younger than 30 months at baseline and receive TRIAC for 96 months at a higher dose compared to TRIAC Trial I. A detailed analysis of this trial’s outcome still needs to be awaited. If an early onset of TRIAC therapy turns out to alleviate neurological abnormalities, patients with MCT8 deficiency should ideally be identified as early as possible after birth. Unfortunately, neonatal screening for TSH or T4 levels in dried blood samples is not suitable for AHDS, as these parameters are in the normal range at birth. Rather, patients exhibit low rT3 concentrations and thus measurement of rT3 and T3/rT3 has been suggested as a suitable early biomarker to disclose MCT8 deficiency (57).

Nonetheless, even if patients are identified directly after birth and receive immediate treatment with TRIAC or other thyromimetic substances, one cannot expect to achieve completely normal neurocognitive development as seen in Mct8/Oatp1c1 DKO. Since, in comparison to the murine brain, the human CNS is more mature at birth, TH-dependent neural maturation processes are certainly compromised already prenatally in MCT8 deficiency, and those impairments will putatively not benefit from early postnatal treatment initiation (11, 14).

## Phenylbutyrate treatment

Several AHDS-related MCT8 mutations do not abolish TH transport capacity *per se* but rather prevent proper protein folding and cell surface translocation. A prominent example is the pathogenic MCT8delF501 mutation that compromises MCT8 protein stability, while TH transport activity of residual MCT8 cell surface protein is retained (58). For these patients, treatment with chaperones that increase the abundance of functional MCT8 protein on the cellular surface may be envisaged. 4-Phenylbutyrate (PB), a FDA-approved drug for the treatment of urea cycle disorder (59), as well as its salt sodium phenylbutyrate (NaPB), have been shown to enhance protein stability and increase cell-surface expression of several transmembrane proteins harboring missense mutations, among them the cystic fibrosis transmembrane conductance regulator (CFTR) (60). In several *in vitro* studies, mutated MCT8 (harboring distinct missense mutations) also showed increased cell surface abundance of MCT8 in the presence of NaPB. Intriguingly, NaPB treatment of induced brain microvascular endothelial cells expressing either the pathogenic MCT8-P321L mutation or a corrected MCT8 not only increased MCT8 protein levels but also stimulated T3 uptake in the presence of an MCT8 inhibitor. The latter finding suggests that the expression of other TH transporters is induced by NaPB treatment as well (61). A NaPB-induced increase in T3 uptake was also reported in patient-derived fibroblast cultures (62). As this rise was independent of functional MCT8, NaPB appears to have a rather non-MCT8 specific stimulatory impact on gene expression. Although further studies are required to dissect the molecular mechanism underlying NaPB-stimulated TH transport, these current observations fit well with the established activity of NaPB as a histone deacetylase inhibitor that can upregulate the expression of epigenetically silenced genes (59).

Only recently have the first clinical attempts to evaluate PB as a treatment for AHDS been reported (63, 64). Application of a high dose of NaPB or glycerol-PB indeed improved thyroid function test parameters. Yet, during the overall treatment period of 13 months, all three MCT8 patients experienced episodes with elevated liver enzymes, and due to these adverse effects, the application of PB had to be temporarily interrupted. Whether long-term PB treatment would ultimately ameliorate neurocognitive impairments still needs to be addressed. Further, pre-clinical studies assessing PB effects on neurodevelopmental parameters would be highly informative as they would allow a direct comparison of PB action with already tested thyromimetic substances.

## Gene therapy with AAV-MCT8 vector constructs

Gene therapy exploiting adeno-associated virus (AAV) – vector constructs has emerged as a promising approach to treat monogenetic disorders, and therefore, it has also been considered for MCT8 deficiency. By delivering MCT8-expressing AAV constructs into the CNS that efficiently transduce neural target cells, one would not only restore MCT8-dependent TH transport but also enable the cellular transmembrane passage of other putative substrates of MCT8. In a first proof-of-concept study, an AAV9-MCT8 construct was applied to neonatal Mct8 KO mice by either i.v. or i.c.v. administration (65). Though i.c.v. delivery resulted in MCT8 protein expression, an increased brain T3 content could only be achieved upon injecting the AAV intravenously. This latter finding can be explained by much stronger MCT8 expression in choroid plexus structures upon i.v. delivery of the constructs. It also underscores the relevance of a functional MCT8 transporter in brain barrier cells as a gatekeeper for TH entrance.

The impact of AAV9-MCT8 application on brain function was subsequently assessed in juvenile Mct8/Oatp1c1 DKO mice that received the construct via i.v. injection at P30 (66). This treatment partially restored brain T3 content and improved behavioral performance, although cellular expression pattern of MCT8 was not evaluated. It also remains to be clarified whether neural maturation deficits can be prevented upon AAV9-MCT8 injection in neonatal Mct8/Oatp1c1 DKO mice.

As AAV9 is established to primarily target neurons, another AAV-MCT8 virus construct was generated that specifically transduces BBB endothelial cells (67). Application of this so-called AAV-BR1-MCT8 in neonatal Mct8/Oatp1c1 DKO mice indeed corrected many brain parameters and fully restored locomotor performance, thereby achieving similar beneficial effects as seen under TRIAC treatment (68). AAV-BR1-MCT8 delivery was also effective in juvenile Mct8/Oatp1c1 DKO mice, where it also increased brain T3 content and signaling and restored behavioral performance. Yet, AAV-BR1-MCT8 treated animals still displayed deranged TH parameters and exhibited a thyrotoxic state in peripheral tissues. Thus, in future studies, it may be worthwhile to combine an AAV-BR1-MCT8 application with a low dose of TRIAC or DITPA administration to maximize therapeutic effects.

Despite these very encouraging preclinical data, further studies are required before applying these vector constructs to patients. The tropism of AVV-BR1 toward BBB endothelial cells is probably restricted to the mouse as AAV-BR1 was not effective in rats (69). Only recently, a novel AAV9 capsid variant (AAV-X1) has been developed that exhibits a significant improvement in targeting the CNS compared to its parent AAV9 and also shows high specificity toward brain endothelial cells with a broad tropism in primates (70). It is, therefore, reasonable to await the generation of such novel constructs and to test them first in preclinical models of AHDS.

## Prenatal treatment approaches

In families with a history of AHDS, MCT8 patients may be diagnosed already prenatally, and for those individuals, even intrauterine treatment might be an option as brain damage appears to occur already during fetal stages (14). A male fetus carrying the same MCT8 mutation as his older brother received weekly 500 µg L-T4 via intra-amniotic injections from gestational week 18 until spontaneous preterm delivery at gestational week 35 (71). After birth, treatment was continued by L-T4/PTU application. Although in-depth neurological assessment confirmed severely impaired neurodevelopment in both patients, the younger brother presented higher achievements in receptive language, problem-solving, and gross and fine motor functions. Likewise, MRI analysis at 6 months of age showed improved brain myelination in the treated patient compared to the untreated brother, indicating a partial rescue of the neurodevelopmental abnormalities. This case report underscores the requirement of MCT8-facilitated TH transport for prenatal brain maturation.

*In vitro* studies further support a critical function of MCT8 during human cortex development. MCT8-deficient cerebral organoids derived from human induced pluripotent stem cells exhibit smaller rosettes and thinner cortices, indicating that MCT8 is already required in neural precursor cells during early fetal development (72). Of note, acute treatment of these organoids with either DITPA or TRIAC restored normal expression of T3-responsive genes. Yet, it remains to be investigated whether long-term incubation of organoids with these thyromimetics results in a normal neural proliferation and differentiation pattern. Eventually, DITPA treatment of pregnant women carrying an affected fetus can also be considered as DITPA has less impact on the maternal HPT axis compared to TRIAC. In fact, a clinical study has recently been initiated to evaluate this treatment option (NCT04143295).

## Conclusions

Despite numerous strategies developed and tested in recent years, designing an optimal treatment for MCT8 deficiency remains challenging. Peripheral thyrotoxicosis caused by high serum T3 levels can be mitigated by a combination of L-T4 and PTU. The application of TRIAC and DITPA also effectively suppresses the elevated serum T3 and ameliorates many peripheral parameters of thyrotoxicosis without any major adverse effects. Treatment with PB improves the deranged TH parameters as well, but also exerts adverse liver effects. Unfortunately, none of these interventions has so far resulted in profound beneficial effects on the neurological outcome. This may be partially due to a missed time window of opportunity, as any intervention that aims to improve brain parameters should ideally be initiated as early as possible. Future studies will reveal whether a gene therapy that includes the delivery of functional MCT8 into and across the brain vasculature can pave the way to at least attenuate the severity of neurodevelopmental and neurocognitive deficits.

## Declaration of interest

The University Hospital Essen receives royalties from Rare Therapeutics, and HH serves on the scientific advisory board of Egetis Therapeutics. None of the authors benefits personally from any royalties.

## Funding

This work was supported by grants from the DFG to SM (CRC/TR296- P19) and HH (CRC/TR296- P01, P09). Funding was further provided by the BMBF (01GM1401) and the Sherman Family to HH.

## Author contribution statement

BM: writing – review and editing; generation of figures; HH: writing – original draft; generation of figures; SM: writing – review and editing; generation of figures.
